# Efficacy and Safety of Praziquantel in Preschool-Aged Children in an Area Co-Endemic for *Schistosoma mansoni* and *S. haematobium*

**DOI:** 10.1371/journal.pntd.0001917

**Published:** 2012-12-06

**Authors:** Jean T. Coulibaly, Yve K. N'Gbesso, Stefanie Knopp, Jennifer Keiser, Eliézer K. N'Goran, Jürg Utzinger

**Affiliations:** 1 Department of Epidemiology and Public Health, Swiss Tropical and Public Health Institute, Basel, Switzerland; 2 University of Basel, Basel, Switzerland; 3 Unité de Formation et de Recherche Biosciences, Université Félix Houphouët-Boigny, Abidjan, Côte d'Ivoire; 4 Centre Suisse de Recherches Scientifiques en Côte d'Ivoire, Abidjan, Côte d'Ivoire; 5 Centre de Santé Rural d'Azaguié, Departement d'Agboville, Azaguié, Côte d'Ivoire; 6 Department of Medical Parasitology and Infection Biology, Swiss Tropical and Public Health Institute, Basel, Switzerland; The George Washington University Medical Center, United States of America

## Abstract

**Background:**

In sub-Saharan Africa the recommended strategy to control schistosomiasis is preventive chemotherapy. Emphasis is placed on school-aged children, but in high endemicity areas, preschool-aged children are also at risk, and hence might need treatment with praziquantel. Since a pediatric formulation (e.g., syrup) is not available outside of Egypt, crushed praziquantel tablets are used, but the efficacy and safety of this treatment regimen is insufficiently studied.

**Methodology:**

We assessed the efficacy and safety of crushed praziquantel tablets among preschool-aged children (<6 years) in the Azaguié district, south Côte d'Ivoire, where *Schistosoma mansoni* and *S. haematobium* coexist. Using a cross-sectional design, children provided two stool and two urine samples before and 3 weeks after treatment. Crushed praziquantel tablets, mixed with water, were administered at a dose of 40 mg/kg. Adverse events were assessed and graded 4 and 24 hours posttreatment by interviewing mothers/guardians.

**Principal Findings:**

Overall, 160 preschool-aged children had at least one stool and one urine sample examined with duplicate Kato-Katz thick smears and a point-of-care circulating cathodic antigen (POC-CCA) cassette for *S. mansoni*, and urine filtration for *S. haematobium* diagnosis before and 3 weeks after praziquantel administration. According to the Kato-Katz and urine filtration results, we found high efficacy against *S. mansoni* (cure rate (CR), 88.6%; egg reduction rate (ERR), 96.7%) and *S. haematobium* (CR, 88.9%; ERR, 98.0%). POC-CCA revealed considerably lower efficacy against *S. mansoni* (CR, 53.8%). Treatment was generally well tolerated, but moderately severe adverse events (i.e., body and face inflammation), were observed in four *Schistosoma* egg-negative children.

**Conclusions/Significance:**

Crushed praziquantel administered to preschool-aged children at a dose of 40 mg/kg is efficacious against *S. mansoni* and *S. haematobium* in a co-endemic setting of Côte d'Ivoire. Further research is required with highly sensitive diagnostic tools and safety must be investigated in more depth.

**Trial Registration:**

Controlled-Trials.com ISRCTN53172722

## Author Summary

Schistosomiasis is a parasitic worm infection that plagues more than 200 million people in the developing world, particularly in sub-Saharan Africa. The current strategy to control schistosomiasis is to regularly administer the deworming drug praziquantel to school-aged children. Younger children before reaching school-age are not included in these deworming campaigns, because they are considered at low risk of schistosomiasis, and because the amount of available data to evaluate the safety of praziquantel in young children is insufficient. We conducted a study in two villages in southern Côte d'Ivoire and examined the stool and urine of more than 250 children (<6 years) for schistosome eggs and antigens. Children were treated with crushed praziquantel tablets (40 mg/kg) and the efficacy of this treatment was determined 3 weeks after treatment. The safety of the treatment was assessed by interviewing mothers of treated children for adverse events (e.g., abdominal pain, diarrhea, and headache). Complete data records were available for 160 children. Praziquantel cleared most of the infections. The treatment was generally well tolerated, but we observed four children who were not infected at the baseline survey who developed face and body inflammation that required close supervision by the study physician.

## Introduction

Schistosomiasis is still a major public health problem in many parts of the developing world, especially in sub-Saharan Africa [1]–[5]. Indeed, more than 200 million people are infected, with about half of them suffering from morbid sequelae, including hematuria, dysuria, nutritional deficiencies, anemia, and delayed physical and cognitive development [1], [5]–[9]. The anthelmintic drug praziquantel is the cornerstone for morbidity control with millions of people treated every year [10]–[14]. Morbidity control is emphasized since the mid-1980s, and this strategy has been reinforced in 2001 by the World Health Assembly (WHA) resolution 54.19, which urged member states to regularly de-worm at least 75% and up to 100% of school-aged children at risk of schistosomiasis and soil-transmitted helminthiasis [10], [15].

Preschool-aged children (individuals below the age of 5–6 years) are currently excluded from preventive chemotherapy control campaigns. The main reasons for this exclusion are that preschool-aged children are believed to be at low risk of schistosomiasis [16], and that there is insufficient data documenting the safety and efficacy of praziquantel in this age group [17], [18]. However, recent studies carried out in different parts of East and West Africa showed that in high endemicity areas a considerable proportion of preschool-aged children are already infected with *Schistosoma*, and hence treatment might need to be extended to younger age groups in such high-risk areas [19]–[29].

With regard to morbidity control of schistosomiasis using praziquantel, it is important to note that an appropriate pediatric formulation for treating preschool-aged children is currently not available outside of Egypt (i.e., praziquantel syrup, Epiquantel, manufactured by the Egyptian International Pharmaceutical Industries Co. A.R.E., Cairo, Egypt). Hence, a common approach in high endemicity areas is to use praziquantel tablets (600 mg), crush them between two spoons, mix with water or fruit juice, and then administer orally to preschool-aged children at a dose of 40 mg/kg [17], [23], [24]. Recent studies using Epiquantel in preschool-aged children revealed similar efficacy than crushed praziquantel tablets [28], [30].

The study reported here was designed to assess the efficacy and safety of crushed praziquantel tablets in preschool-aged children (<6 years) in an area where *Schistosoma mansoni* and *S. haematobium* coexist [31], [32]. Our findings, along with other investigations pertaining to the epidemiology of schistosomiasis in preschool-aged children, may be useful to further optimize the control of schistosomiasis in a currently neglected age group.

## Methods

### Ethics Statement

Ethical approval was granted by the Ministry of Health and Public Hygiene of Côte d'Ivoire (reference no. 4248/2010/MSHP/CNER). The trial is registered with ClinicalTrial.gov (identifier: ISRCTN 53172722) and the protocol is available as Supporting Information (Protocol S1). Local authorities of the Azaguié district were informed about the purpose, procedures, and potential risks and benefits of the study. In the absence of recent census data, an exhaustive door-to-door survey was carried out to identify all preschool-aged children (<6 years) in the two selected villages. Parents or guardians of eligible children were informed about the objectives of the study and asked to provide written informed consent on behalf of their children. Only those preschool-aged children who had written informed consent from their parents/guardians were included.

Participation was voluntary and parents/guardians could withdraw their child from the study anytime without further obligation. At the end of the study, anthelmintic drugs (i.e., praziquantel against schistosomiasis and albendazole against soil-transmitted helminthiasis) were offered free of charge to all community members.

### Study Area and Population

The study was conducted between June and November 2011 in two villages located in the district of Azaguié, southern Côte d'Ivoire: Azaguié Makouguié (geographical co-ordinates 05°37′33″N latitude, 04°09′04″W longitude) and Azaguié M'Bromé (05°39′42″N, 04°08′38″W). Recent studies have shown that *S. mansoni* and *S. haematobium* are co-endemic in Azaguié [31]–[33]. Villagers are mainly engaged in subsistence farming. Both villages lack access to clean water and improved sanitation.

### Study Design and Sample Size

We pursued an intervention study (i.e., praziquantel administration), including children of both sexes below the age of 6 years. Children's infection with *S. mansoni* and *S. haematobium* was assessed during a baseline cross-sectional survey and again 3 weeks posttreatment using standardized, quality-controlled methods. The STROBE checklist is available as Supporting Information (Checklist S1).

With the overarching goal to further the understanding of the epidemiology, diagnosis, and control of schistosomiasis in preschool-aged children, we aimed at a sample of about 200 individuals, as recommended by statistical textbooks in health studies [34]. Our population census carried out in June 2011 revealed 367 preschool-age children. We assumed that the prevalence of *S. mansoni* in preschool-aged children would be around 20% (i.e., a quarter of the 80% *S. mansoni* infection prevalence observed in school-aged children in Azaguié in 2010 [31]) and that about 70% of the preschool-aged children would comply (lower rate than among school-aged children due to the difficulty to obtain biological samples in this younger age group). Aiming for a precision of 5%, we finally decided to include all 367 registered preschool-aged children.

### Inclusion Criteria

We adhered to the following inclusion criteria: (i) preschool-aged children (<6 years); (ii) written informed consent by parents/guardians; (iii) submission of at least one sufficiently large stool sample for duplicate Kato-Katz thick smears, and one urine sample for a 10 ml filtrate and a single point-of-care circulating cathodic antigen (POC-CCA) cassette test; (iv) no abnormal medical condition, as judged by the study physician on the day of the treatment; (v) no recent anthelmintic treatment (within the past 4 weeks) according to a parental questionnaire; and (vi) no participation in any other clinical trial.

### Parasitological and Clinical Examinations

The door-to-door census carried out in June 2011 to establish up-to-date census data generated lists of preschool-aged children, including their name, age, sex, and geographical coordinates of the household. Mothers/guardians of the preschool-aged children were provided with plastic containers labeled with unique identifiers (IDs) and they were asked to obtain a fresh stool and urine sample of their child. Stool and urine samples were collected at any time of the day due to the difficulty of collecting biological samples in this age group. Our aim was to obtain two stool and two urine samples over two consecutive days from each participating child.

### Laboratory Procedures

Stool and urine samples were transferred to a nearby laboratory in Azaguié town and worked up on the day of collection. For the diagnosis of *S. mansoni* and soil-transmitted helminths, duplicate Kato-Katz thick smears were prepared from each stool sample (i.e., quadruplicate Kato-Katz thick smears per child), using 41.7 mg templates [35]. The Kato-Katz thick smears were allowed to clear for at least 30 min before examination under a microscope by experienced laboratory technicians. The number of *S. mansoni* and soil-transmitted helminth eggs were counted and recorded for each species separately.

For the diagnosis of *S. haematobium*, urine samples were subjected to a filtration method, as described elsewhere [36]. Briefly, a single filtration was performed with each urine sample (i.e., two urine filtrations per child over two consecutive days). Urine samples were vigorously shaken and 10 ml pressed through a small-meshed filter (aperture: 30 µm) and a drop of Lugol's solution was added on the filter paper, which was then placed onto a microscope slide. Slides were examined under a microscope and the number of *S. haematobium* eggs counted by experienced technicians.

For quality control, 10% of the Kato-Katz thick smears and the urine filter slides were re-examined by a senior technician. In case of disagreement, the results were discussed with the concerned technician and discordant slides re-read until agreement was reached.

An additional approach for the diagnosis of *S. mansoni* was employed, namely a POC-CCA cassette, that is based on a commercially available lateral flow immuno-chromatographic test [31]. The POC-CCA cassette (batch 33112) was performed according to the manufacturer's instructions. In brief, a drop of urine was added to the well and once fully absorbed a drop of buffer was added. The tests were read within 20–25 min. In case the control band failed to develop, the test was considered invalid and the respective urine sample retested with a new POC-CCA. Valid tests were scored as negative or positive, the latter stratified into trace (very light color band), 1+, 2+, and 3+ according to the visibility of the color reaction. The tests were scored independently by two investigators. In case of conflicting results, a third investigator was consulted, and the results discussed until agreement was reached [31].

### Praziquantel Treatment and Monitoring of Adverse Events

Children were treated with crushed praziquantel tablets (600 mg; Biltricide, Bayer) at a dose of 40 mg/kg [10]. Children were weighed using an electronic balance (Evolis; Rumily, France). The appropriate number of praziquantel tablets (e.g., half a tablet for a child weighing 7–8 kg) were crushed between two spoons, mixed with tap water in a clean soup spoon before oral administration. Treatment was given by the mothers/guardians of the children under close supervision of trained medical personnel. Children were closely monitored by medical staff for 4 hours. In case vomiting occurred within 1 hour after treatment, a second dose of praziquantel was administered.

Treatment-related adverse events were assessed 4 and 24 hours posttreatment. Mothers/guardians were asked to report unusual behavior of their children since drug intake, and whether any of the following adverse events occurred: abdominal pain, allergic reaction, diarrhea, dizziness, fatigue, fever, headache, nausea, and vomiting. Adverse events were graded (i.e., light, moderate, severe, or life threatening), as described elsewhere [37].

### Treatment Efficacy Evaluation

Three weeks after praziquantel administration, stool and urine samples were collected again, using the same procedures. Treatment efficacy was determined by means of cure rate (CR, percentage of children positive at the pretreatment cross-sectional survey who became egg-negative 3 weeks after treatment, as assessed by the Kato-Katz technique for *S. mansoni* and urine filtration for *S. haematobium*) and egg reduction rate (ERR, reduction in the group's geometric mean fecal egg count for *S. mansoni* or the group's geometric mean *S. haematobium* egg count in 10 ml of urine comparing the before and after treatment situation).

### Statistical Analysis

Data were double entered into an Excel spreadsheet, transferred into EpiInfo version 3.2 (Centers for Disease Control and Prevention; Atlanta, USA) and cross-checked. Statistical analyses were done with Stata version 10 (Stata Corp.; College Station, USA). Preschool-aged children who had at least one stool sample examined with duplicate Kato-Katz thick smears, a single POC-CCA cassette for *S. mansoni* diagnosis, and one urine sample subjected to a filtration method for *S. haematobium* diagnosis before and after treatment were included in the final analysis (per-protocol). Continuous data (e.g., schistosome egg counts) are presented as geometric mean, whereas dichotomous data (e.g., presence or absence of an infection) are presented as proportion.

Infection intensities were stratified according to cut-offs proposed by the World Health Organization (WHO) [10]. There are three intensity classes for *S. mansoni*: (i) light (i.e., 1–99 eggs/gram of stool (EPG)); (ii) moderate (100–399 EPG); and (iii) heavy (≥400 EPG). *S. haematobium* infections were categorized as light (1–49 eggs/10 ml of urine) and heavy (≥50 eggs/10 ml of urine).

## Results

### Adherence and Population Characteristics

**Figure 1:**
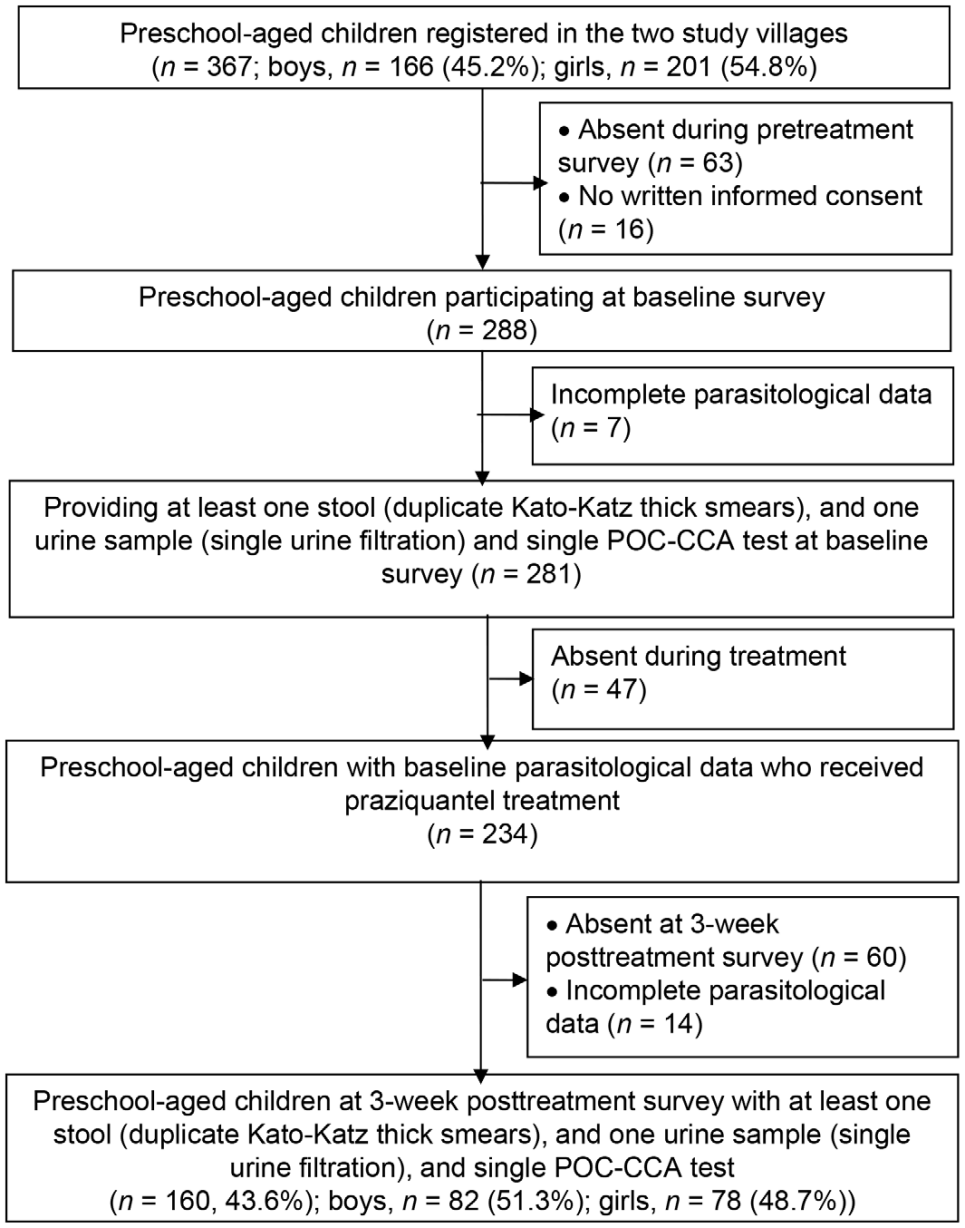
Flow chart and study adherence. The study was carried out in the villages of Azaguié Makouguié and Azaguié M'Bromé in south Côte d'Ivoire, between June and November 2011.

Figure 1 shows the adherence of preschool-aged children to the study protocol. The village census revealed 367 children aged below 6 years, all of whom were invited to participate. Sixty-three children were absent during the baseline survey and 16 had no written informed consent by their parents/guardians. From the 288 children participating at the baseline cross-sectional survey, seven were excluded due to incomplete parasitological data (e.g., insufficiently large stool sample for duplicate Kato-Katz thick smears). Among the remaining 281 children, 234 were administered crushed praziquantel. Three weeks posttreatment, we were able to re-examine at least one sufficiently large stool and urine sample from 160 children.

Our final study cohort consisted of 82 (51.3%) boys and 78 girls with an average age of 3.2 years (range: 5 months to 5 years). Boys were slightly younger than girls (average, 3.0 years; 95% confidence interval (CI), 2.7–3.3 years *versus* average, 3.3 years; 95% CI, 3.0–3.6 years).

### Baseline Characteristics

Table 1 shows the pretreatment *S. mansoni* and *S. haematobium* infections, stratified by children's sex and diagnostic approach. According to at least duplicate Kato-Katz thick smears, 35 children of our per-protocol population (21.9%) were found *S. mansoni*-positive, with a geometric mean infection intensity of 1.2 EPG (Table 2). According to the POC-CCA results, there were 128 *S. mansoni* infections (80.0%) when considering ‘trace’ results as positive, and 78 (48.7%) considering ‘trace’ results as negative. With regard to *S. haematobium*, the urine filtration method revealed 18 infections (11.2%). The geometric mean infection intensity was 1.0 eggs/10 ml of urine. Eleven children (6.9%) were co-infected with *S. mansoni* and *S. haematobium*.

**Table 1:** Baseline prevalence of *S. mansoni* and *S. haematobium*, stratified by sex and diagnostic approach.

Species	Diagnostic approach	Sex	No. of children examined	No. (%) of infected children
*S. mansoni*	Kato-Katz	Male	82	19 (23.2)
		Female	78	16 (20.5)
		Total	160	35 (21.9)
	POC-CCA test (incl. trace)	Male	82	68 (82.9)
		Female	78	60 (76.9)
		Total	160	128 (80.0)
	POC-CCA test (excl. trace)	Male	82	44 (53.7)
		Female	78	34 (43.6)
		Total	160	78 (48.7)
*S. haematobium*	Urine filtration	Male	82	9 (10.9)
		Female	78	9 (11.5)
		Total	160	18 (11.2)

The study was carried out in two villages in Azaguié, south Côte d'Ivoire, between June and November 2011. All preschool-aged children who had at least one stool sample examined with duplicate Kato-Katz thick smears and one urine sample subjected to a filtration method before and 3 weeks after praziquantel administration were included (per-protocol analysis).

POC-CCA, point-of-care circulating cathodic antigen.

**Table 2:** *S. mansoni* and *S. haematobium* cure and egg reduction rates, as assessed 3 weeks after praziquantel treatment, stratified by diagnostic approach.

Species	Diagnostic approach	No. (%) of infected children		CR in % (95% CI)	Geometric mean infection intensity (95% CI)		ERR in %
		Baseline	Follow-up		Baseline	Follow-up	
*S. mansoni*	Kato-Katz	35 (21.9)	4 (2.5)	88.6 (73.3–96.8)	1.2 (0.9–1.4) EPG	0.04 (0–0.09) EPG	96.7
	POC-CCA test (incl. trace)	128 (80.0)	79 (49.4)	38.3 (29.8–47.3)	NA	NA	NA
	POC-CCA test (excl. trace)	78 (48.7)	36 (22.5)	53.8 42.2–65.2)	NA	NA	NA
*S. haematobium*	Urine filtration	18 (11.2)	2 (1.3)	88.9 (65.3–98.6)	1.0 (0.9–1.1) eggs/10 ml urine	0.02 (0–0.06) eggs/10 ml urine	98.0

The study was carried out in two villages in Azaguié, south Côte d'Ivoire, between June and November 2011, focusing on preschool-aged children.

CI, confidence interval; CR, cure rate; EPG, eggs/gram of stool; ERR, egg reduction rate; NA, not applicable; POC-CCA, point-of-care circulating cathodic antigen.

The Kato-Katz technique also allows detection of soil-transmitted helminth eggs. The observed prevalence of *Trichuris trichiura*, hookworm and *Ascaris lumbricoides* was 10.9%, 5.9% and 3.8%, respectively.

### Praziquantel Efficacy

Table 2 summarizes CR and ERR, stratified by diagnostic approach. According to the Kato-Katz technique, at the 3-week posttreatment follow-up, four children (2.5%) were identified with *S. mansoni* eggs in their stool with a geometric mean infection intensity of 0.04 EPG. The CR and ERR was 88.6% and 96.7%, respectively. The POC-CCA results only allowed estimating CR. Including or excluding ‘traces’ as positive results, revealed 79 and 36 *S. mansoni*-infected children, respectively, at the 3-week posttreatment follow-up. The respective CRs were 38.3% and 53.8%.

With regard to *S. haematobium*, two children (1.3%) had a positive urine filtration at the 3-week posttreatment follow-up with a geometric mean infection intensity of 0.02 eggs/10 ml of urine. The CR and ERR were 88.9% and 98.0%, respectively.

**Figure 2:**
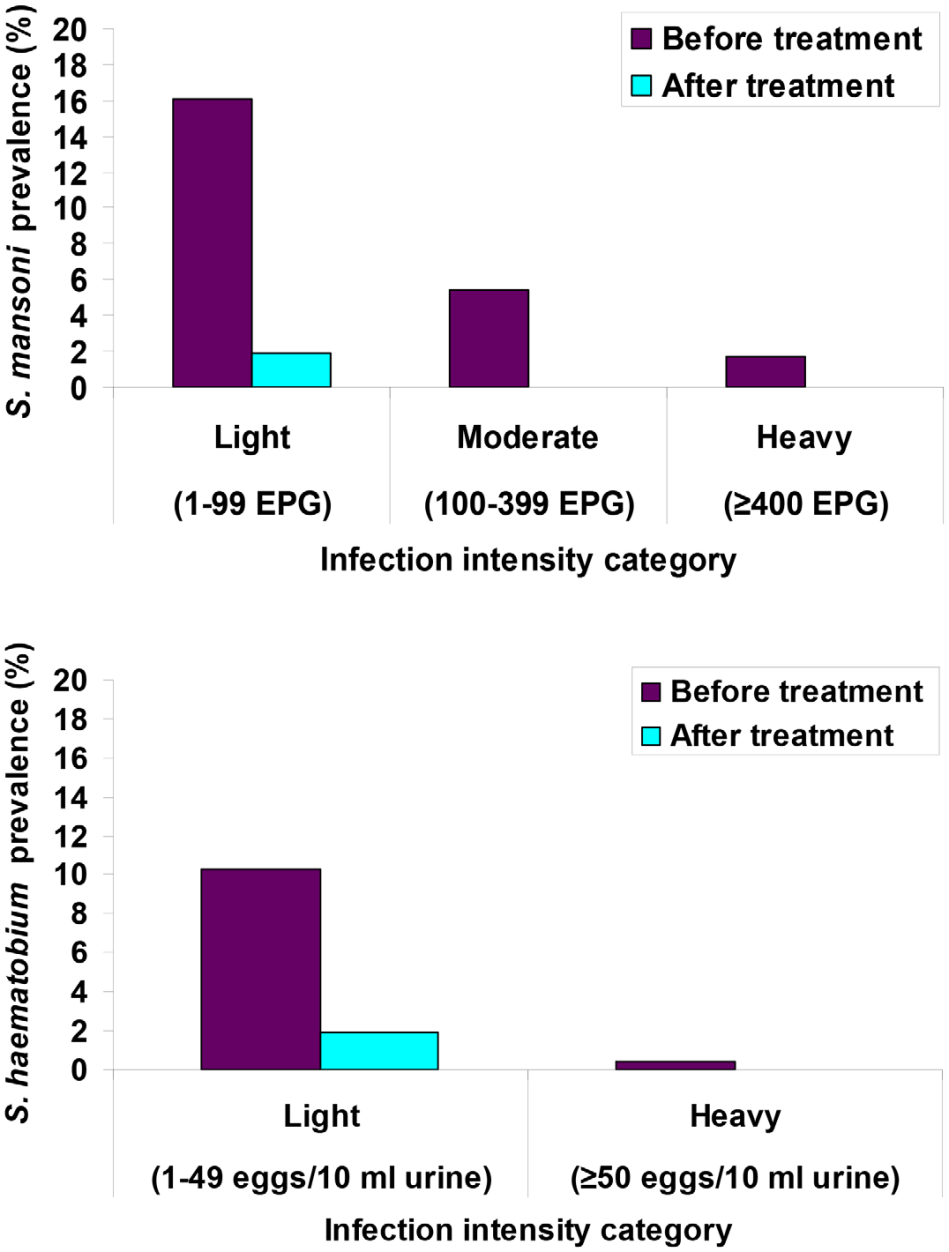
*S. mansoni* and *S. haematobium* infection status of preschool-aged children before and 3 weeks after praziquantel administration. Parasitological results are based on duplicate Kato-Katz thick smear examinations (for *S. mansoni*) and urine filtration (for *S. haematobium*).

Figure 2 shows infection intensity categories of *S. mansoni* and *S. haematobium* at the baseline and posttreatment surveys. At baseline, among 35 *S. mansoni*-infected children, 23, nine and three children had light, moderate and heavy infections, respectively. With regard to *S. haematobium*, 17 children and one child were lightly or heavily infected, respectively. At the 3-week posttreatment follow-up all children who were still *Schistosoma*-positive had light infections.

### Adverse Events

Table 3 shows the incidence of adverse events 4 and 24 hours after praziquantel administration among the 234 treated children. Overall, 43 children reported to have adverse events. Among these children, 12 (27.9%) were coinfected with *S. mansoni* and *S. haematobium*, 17 (39.5%) had *S. mansoni* single infection, no child was infected with *S. haematobium* only, and 14 children (32.6%) were not infected at all. We stratified adverse events into two independent groups. The first group designated children with adverse events reported within 4 hours posttreatment and the second group was made up by children with adverse events reported by their mothers/guardians 24 hours posttreatment. Most of the adverse events were observed within the first 4 hours after treatment (*n* = 32, 74.4%), including abdominal pain (*n* = 7), diarrhea (*n* = 6), nausea (*n* = 5), vomiting (*n* = 4), dizziness (*n* = 3), fever (*n* = 2), fatigue (*n* = 2), face and body inflammation (*n* = 2), and headache (*n* = 1). More than one adverse event was observed in three (abdominal pain and diarrhea), two (vomiting and nausea) and one (fever and dizziness) children within 4 hours posttreatment. Twenty-four hours posttreatment, 11 (25.6%) children reported adverse events, including diarrhea (*n* = 3), abdominal pain (*n* = 2), fatigue (*n* = 2), face and body inflammation (*n* = 2), dizziness (*n* = 1), and headache (*n* = 1). At this time point, none of the children reported multiple adverse events.

**Table 3:** Adverse events 4 and 24 hours after administration of crushed praziquantel tablets (*n* = 234).

Adverse event	Severity	Incidence (%)	
		4 hours	24 hours
Abdominal pain	Light	7 (2.9)	2 (0.9)
Diarrhea		6 (2.6)	3 (1.3)
Nausea		5 (2.1)	0
Vomiting		4 (1.7)	0
Dizziness		3 (1.3)	1 (0.4)
Fatigue		2 (0.9)	2 (0.9)
Fever		2 (0.9)	0
Headache		1 (0.4)	1 (0.4)
Body and face inflammation	Moderate	2 (0.9)	2 (0.9)
Total		32	11

The study was carried out in two villages in Azaguié, south Côte d'Ivoire between June and November 2011, focusing on preschool-aged children.

Adverse events were considered of light severity, with the only exception of face and body inflammation that was graded as a moderately severe. Mothers/guardians with children experiencing body and face inflammation sought advice from the study physician who assured the mothers that this adverse event is transient and self-limiting. Indeed, within 24 hours, children's conditions resolved to normal. The four children complaining of body and face inflammation were all *Schistosoma* egg-negative according to Kato-Katz and urine filtration results, but one had a positive POC-CCA test results (1+).

## Discussion

The anthelmintic drug praziquantel is the cornerstone for morbidity control due to schistosomiasis [2], [10]–[14]. Emphasis is placed on school-aged children, whereas preschool-aged children (individuals below the age of 5–6 years) are usually excluded from preventive chemotherapy. However, in highly endemic areas, a considerable amount of preschool-aged children is already affected by schistosomiasis [19]–[29]. For that reason, there is ongoing discussion whether preventive chemotherapy with praziquantel should be extended to preschoolers [17], [24], [25], [28]–[30], [38], [39]. However, absorption and metabolism of drugs are age-dependent [18] and it is not well understood whether the developmental changes in the physiology during biological maturation from newborns to adolescence influence the efficacy and toxicity of praziquantel. We assessed the efficacy and safety of crushed praziquantel among preschool-aged children in an area of south Côte d'Ivoire where *S. mansoni* and *S. haematobium* coexist [31], [32].

Our study confirms that preschool-aged children are at risk of schistosomiasis. Indeed, we found that more than 20% of the children before their sixth birthday were infected with either *S. mansoni* or *S. haematobium*, or both species concurrently (7%). Using a commercially available POC-CCA cassette test, more than half of the children showed positive *S. mansoni* antigen reactions. Our study reveals that crushed praziquantel (40 mg/kg) administered to preschool-aged children is highly efficacious with CRs around 90% and ERRs above 95%, when standard diagnostic methods (Kato-Katz for *S. mansoni* and urine filtration for *S. haematobium*) were used.

There are two limitations of our study worth highlighting. First, we initially aimed to obtain two stool samples (for quadruplicate Kato-Katz thick smears) and two urine samples (for duplicate urine filtration and duplicate POC-CCA cassette test) from each participant before and after praziquantel administration. However, it proved difficult to obtain multiple stool and urine samples in this age group, and hence we finally included preschool-aged children who had at least duplicate Kato-Katz thick smears and a single urine filtration before and after treatment. In view of this sampling effort and diagnostic approach, it is clear that we have missed some infections, particularly those of light intensity. Second, a considerable proportion of preschool-aged children were lost to follow-up. However, comparing our per-protocol population (*n* = 160) with children with incomplete parasitological data, who were absent at treatment, or lost to follow-up (*n* = 121), we found comparable prevalence estimates and infection intensities with *S. mansoni* and *S. haematobium*.

In spite of the aforementioned limitations, our investigation provides new insight into the efficacy and safety of praziquantel among a neglected population group in a *S. mansoni*-*S. haematobium* co-endemic area. Our findings support previous studies conducted in other parts in Africa. For example, a recent study carried out in preschool-aged children in Uganda, using quadruplicate Kato-Katz thick smears (two stool samples, each examined by duplicate Kato-Katz), reported a slightly lower efficacy against *S. mansoni* (CR, 80.2%; ERR, 87.9%) [17]. Mutapi and colleagues in a study done in Zimbabwe with children aged 1–5 years found high CR (92%) and very high ERR (99%) of crushed praziquantel against *S. haematobium* [40]. Of considerable concern are recent findings from Ugandan preschool-aged children, as the overall CR among 305 *S. mansoni* egg-patent individuals was only 56.4%, with particularly low CR observed in preschoolers with a history of previous praziquantel treatments (CR 41.7%) [29]. It should also be noted that the process of crushing praziquantel tablets is time consuming, and hence poses a challenge for large-scale control programs. It is encouraging to note that efforts are under way to develop a pediatric formulation of praziquantel, for example oro-dispersable tablets or minitablets that might be more convenient to administer to small children [30].

In our study, we not only used standard diagnostic tests (i.e., Kato-Katz and urine filtration) but also a more recently developed and now commercially available POC-CCA cassette applied to urine for the diagnosis of *S. mansoni* (Rapid Medical Diagnostics, Pretoria, South Africa). Considering POC-CCA results from the pre- and posttreatment surveys, including or excluding ‘trace’ results as positive, we found low CRs of 38.0% and 53.8%, respectively. These results are worrying and reasons explaining the differences in CR according to the diagnostic technique might be explained as follows. First, *S. mansoni* eggs might have been missed by the Kato-Katz technique, particularly at the posttreatment follow-up when the remaining positive children had very low infection intensities. Indeed, it is widely acknowledged that the Kato-Katz technique lacks sensitivity in areas characterized by low *S. mansoni* infection intensities, which is common after treatment [41]. Second, perhaps CCA might still be detectable in urine 3 weeks after treatment [42], [43], while the recommended time for *S. mansoni* assessment after treatment is 15–20 days [44]. Third, while children might have been cured from patent *S. mansoni* infection, praziquantel is largely refractory against young developing stages of the worms [12], [13], and hence antigens might still be present in the urines of young children. Fourth, the POC-CCA cassette might lack specificity after praziquantel administration. Further investigations are therefore needed to determine whether or not a POC-CCA cassette can be utilized for determining praziquantel efficacy, including the most appropriate time point posttreatment. Three weeks posttreatment might be too short for assessing praziquantel efficacy in preschool-aged children, but the longer one waits, the higher the risk for confounding factors (e.g., schistosomula fully developed into adult worms, and reinfection).

In our study, we also thoroughly assessed the safety of crushed praziquantel given to preschoolers. We observed similar frequencies of adverse events in preschool-aged children as reported by other groups [26], [40], and as observed in school-aged children [45]–[47]. Recently, Namwanje and colleagues showed that praziquantel alone and in combination with mebendazole in the treatment of *S. mansoni* and soil-transmitted helminths in preschool-aged children showed similar safety profiles [48]. However, in the current study, inflammation of the body and the face was observed in four children (2.5%). Interestingly, these children were *Schistosoma* egg-negative at the baseline survey before drug administration and only one child showed a light positive POC-CCA result (1+). Inflammation of body and face has been observed in previous studies [40], raising concern with regard to the inclusion of preschool-aged children in preventive chemotherapy campaigns, which has been proposed by different authors [17], [24], [25], [29]. While preschool-aged children with a confirmed *Schistosoma* infection must be treated [49], we feel that further research is still required, including development of an appropriate pediatric formulation, dose-finding, detailed pharmacokinetic investigations, and in-depth safety studies, before preventive chemotherapy be extended from the school-aged population to preschoolers [18].

In conclusion, our study documents that preschool-aged children are at risk of schistosomiasis in the Azaguié area, south Côte d'Ivoire, with 7% of our per-protocol population patently infected with *S. mansoni* and *S. haematobium* concurrently. Crushed praziquantel is efficacious against both species in preschool-aged children. In view of unwanted adverse events in non-infected children following praziquantel administration, we suggest that only parasitologically confirmed preschool-aged children should be given praziquantel. New research is needed to accurately determine the frequency and severity of adverse events after praziquantel administration against schistosomiasis in the preschool-aged population. There is a need to develop a safe and user-friendly formulation of praziquantel so that infected children can be treated at an early stage of infection in order to prevent any harmful damage in later life.

## Supporting Information

Protocol S1 **Trial protocol.**
(DOC)

Checklist S1 **STROBE checklist.**
(PDF)

Alternative Language Abstract S1 **Translation of the Abstract into French by Jean T. Coulibaly.**
(DOC)
