# Supplementary material for: Efficacy and Safety of Praziquantel in Preschool-Aged Children in an Area Co-Endemic for Schistosoma mansoni and S. haematobium
Source: PLoS Negl Trop Dis. 2012 Dec 6;6(12):e1917. doi: 10.1371/journal.pntd.0001917 (PMC3516585; doi:10.1371/journal.pntd.0001917)
Supplement: Protocol S1 — Trial protocol. (DOC) [file pntd.0001917.s001.doc]

**Final version: 23/8/2011 (uploaded unto Controlled Clinical Trials)**

**Public Title:**

Epidemiology and control of schistosomiasis in preschool-aged children in Côte d’Ivoire, with particular consideration to the efficacy and safety of crushed praziquantel tablets

**Scientific title:**

Epidemiology and control of schistosomiasis in preschool-aged children in Côte d’Ivoire, with particular consideration to the efficacy and safety of crushed praziquantel tablets

**Acronym**

Schisto-Preschool-Cote d’Ivoire

**Serial number at source**

N/A

**Study Hypothesis:**

1. Praziquantel (administered at 40 mg/kg oral dose using crushed tablets) is efficacious against *S. mansoni* and *S. haematobium* infections in preschool-aged children
2. Praziquantel treatment in preschool-aged children is safe

**Lay summary**

Schistosomiasis (a parasitic worm disease) is endemic in Africa. The current strategy for control is based on repeated deworming (using praziquantel tablets), focussing on school-aged children. However, in highly endemic areas, preschool-aged children might already by affected by the disease. In the absence of a paediatric drug formulation (syrup), praziquantel tablets are crushed and administered to preschool-aged children. We will study the epidemiology of schistosomiasis in two highly endemic areas of Cote d’Ivoire and assess the efficacy and safety of crushed praziquantel tablets in preschool-aged children.

**Ethical approval:**

Comité National d’Ethique et de la Recherche (CNER) in Côte d’Ivoire).

Date of approval: 25 August 2010

Reference no. 4248/2010/MSHP/CNER

**Study design:**

Intervention study with 4-week follow-up

**Countries of recruitment:**

Côte d’Ivoire

**Participant inclusion criteria:**

1. Both males and females, aged ≤72 months
2. Written informed consent by parents or legal guardian
3. Submission of 2 stool samples of sufficient size to prepare duplicate Kato-Katz thick smears from each sample at the baseline survey
4. Submission of 2 urine samples of sufficient amount for urine filtration method at the baseline survey
5. Provision of single finger-prick blood sample for malaria rapid diagnostic test and haemoglobin level assessment
6. Absence of major systemic illnesses, as assessed by medical personnel on the day of treatment

**Participant exclusion criteria:**

1. Children aged >72 months
2. No written informed consent provided by by parents or legal guardian
3. Submission of less than 2 stool samples of sufficient size to prepare duplicate Kato-Katz smears from each sample at the baseline or follow-up survey
4. Submission of less than 2 urine samples of sufficient amount for urine filtration method at the baseline survey
5. No provision of finger-prick blood sample for malaria rapid diagnostic test and haemoglobin level assessment
6. Presence of any abnormal medical condition, as judged by the medical personnel on the day of treatment (e.g. clinical malaria)
7. Recent anthelminthic treatment (within 4 weeks)
8. Participation in other studies

**Anticipated start date:**

25 August 2011

**Anticipated end date:**

15 October 2011

**Target number of participants**:

Approximately 350

**Disease/condition/study domain:**

*S. mansoni* and *S. haematobium* infections

Soil-transmitted helminths

Intestinal protozoa

*Plasmodium*

**Interventions**

Study participants diagnosed with either *S. mansoni*, or *S. haematobium* or both species concurrently will be treated with praziquantel (single 40 mg/kg oral dose, using crushed tablets)

**Primary outcome measure:**

Cure rate and egg reduction rate of *S. mansoni* and *S. haematobium*, determined 3-4 weeks post-treatment by multiple stools sampling using the Kato-Katz method and multiple urine filtration tests

**Secondary outcome measures:**

Frequency and severity of adverse events recorded within 24 hours after drug administration

**Sources of funding**

Rudolf Geigy-Stiftung zu Gunsten des Schweizerischen Tropen- und Public Health-Instituts

Schweizerisches Tropen- und Public Health-Institut

Socinstrasse 57

CH-4051 Basel, Schweiz
