# Supplementary material for: Efficacy and Safety of Praziquantel in Preschool-Aged Children in an Area Co-Endemic for Schistosoma mansoni and S. haematobium
Source: PLoS Negl Trop Dis. 2012 Dec 6;6(12):e1917. doi: 10.1371/journal.pntd.0001917 (PMC3516585; doi:10.1371/journal.pntd.0001917)
Supplement: Alternative Language Abstract S1 — Translation of the Abstract into French by Jean T. Coulibaly. (DOC) [file pntd.0001917.s003.doc]

**Résumé**

***Contexte:*** En Afrique du sud du Sahara la stratégie recommandée pour le contrôle de la schistosomiase est la chimiothérapie préventive. Un accent est mis sur les enfants d’âge scolaire, cependant dans les zones de haute endémicité, les enfants d’âge préscolaire sont aussi à risque, et pourraient avoir besoin du traitement au praziquantel. Puisqu’une formulation (Ex: le sirop) n’est pas disponible en dehors de l’Egypte, les comprimés concassés de praziquantel sont utilisés, mais l’efficacité et la sécurité de ce régime de traitement sont insuffisamment étudiés.

***Méthodologie:*** Nous avons évalué l’efficacité et la sécurité des comprimés concassés de praziquantel chez les enfants d’âge préscolaire (<6 ans) dans le district d’Azaguié, sud de la Côte d’Ivoire, où *Schistosoma mansoni* et *S. haematobium* coexistent. Utilisant le design d’une étude transversale, les enfants ont fourni chacun deux échantillons de selles et d’urine, avant et trois semaines après le traitement. Les comprimés concassés de praziquantel, mixés à l’eau, ont été administrés à la dose de 40 mg/kg de poids corporel. Les effets indésirables ont été évalués en interviewant les mères/tutrices légales, 4 et 24 heures post-traitement et ceux-ci ont été gradés.

***Résultats:*** Dans l’ensemble, 160 enfants d’âge préscolaire ont fourni au moins un échantillon de selles et d’urine. Deux lames de Kato-Katz et un test POC-CCA (détectant les antigènes cathodiques circulants) ont été réalisés en vue du diagnostic de *S. mansoni* tandis qu’une filtration d’urine a été réalisée pour le diagnostic de *S. haematobium* et cela avant et 3 semaines après l’administration du praziquantel. Selon les résultats du Kato-Katz et de la filtration d’urine, une grande efficacité du praziquantel contre *S. mansoni* (taux de guérison (TG), 88.6%; Taux de réduction des oeufs (TRO), 96.7%) et *S. haematobium* (TG, 88.9%; TRO, 98.0%) a été observée. Par contre, le test POC-CCA a révélé une efficacité considérablement faible contre *S. mansoni* (TG, 53.8%). Le traitement a été généralement bien toléré, mais des effets indésirables, modérément sévères (c-à.d, l’inflammation du corps et de la face), ont été observés chez 4 enfants non infectés par la schistosomiase.

***Conclusions:*** Le praziquantel concassé administré aux enfants d’âge préscolaire à la dose de 40 mg/kg de poids corporel est efficace contre *S. mansoni* et *S. haematobium* dans une zone de co-endémicité de la Côte d’Ivoire. Davantage de recherche est exigé, avec des outils de diagnostic hautement sensible, de même, la sécurité du praziquantel doit être investiguée plus en profondeur.
